# Reply to Letter to the Editor in response to: Microvascular endothelial function following cessation of long‐term oral contraceptive pill use

**DOI:** 10.1113/EP091262

**Published:** 2023-05-26

**Authors:** Casey G. Turner, Anna E. Stanhewicz, Karen E. Nielsen, Brett J. Wong

**Affiliations:** ^1^ Department of Kinesiology and Health Georgia State University Atlanta GA USA; ^2^ Molecular Cardiology Research Institute Tufts Medical Center Boston MA USA; ^3^ Department of Health and Human Physiology University of Iowa Iowa City IA USA; ^4^ Department of Population Health Sciences, School of Public Health Georgia State University Atlanta GA USA

1

We thank Kirby et al. for their thoughtful comments regarding our recent work (Turner, Stanhewicz, Nielsen, & Wong, 2023) and are excited that this case report generated thoughts within the scientific community about the vascular physiological effects of oral contraceptive pill (OCP) use and cessation. We would also like to respond to a few of the authors’ major comments, as we believe the interpretation of the presented data is complex, and we had limited space to elaborate on multiple points in the case report format.

We would like to begin by agreeing with Kirby et al. regarding the multiple important positive aspects of OCP use. Despite apparent, positive attributes of OCP, significant gaps in the literature remain (Williams & MacDonald, 2021), particularly regarding long‐term use, post‐OCP use, assessments in the microvasculature and underlying mechanisms. We believe this work (Turner, Stanhewicz, Nielsen, & Wong, 2023) and our other recently published work (Turner, Stanhewicz, Nielsen, Otis et al., 2023) help inform these areas, and further research is important for improved informed decision‐making.

Another major comment from Kirby et al. regards the concurrent interpretation of maximal cutaneous vascular conductance (CVC) alongside percentage maximal CVC (%CVC_max_) values in response to local heating (i.e., plateau, a representative measure of general endothelium‐dependent vasodilatation). We agree that the observed greater maximal CVC during OCP use compared with maximal CVC after cessation of use is intriguing and may, as mentioned, suggest greater endothelium‐independent vasodilatation during OCP use. Kirby et al. also refer to a similar, albeit not statistically significant, observation in our more recently published work assessing cutaneous endothelium‐dependent vasodilatation between women in estimated low hormone phases of the natural menstrual cycle or OCP use and men (Turner, Stanhewicz, Nielsen, Otis et al., 2023). Although it is possible that endothelium‐independent vasodilatation is augmented in women using OCP, it is important to highlight that endothelium‐independent vasodilatation was not a primary outcome variable in either this case report or our other recent work (Turner, Stanhewicz, Nielsen, Otis et al., 2023). Therefore, we were not powered to accurately assess differences in endothelium‐independent vasodilatation. Williams and MacDonald (2021) recently reviewed the literature for the effect of hormonal contraceptive use on smooth muscle cell function and found no effect in either the macro‐ or microvasculature compared with naturally cycling controls or between hormonal phases. Nevertheless, this may be an important area for further investigation.

We are inclined to suggest caution when directly comparing absolute maximal CVC values and their variability. Prior literature has established there is significant inter‐ and intra‐participant variability in red blood cell flux in the skin (Braverman et al., 1990; Johnson et al., 2014). Measurements 2–7 days apart in young, healthy, male participants yielded a coefficient of variation (CV) of 9.8–20.6% in maximal CVC following rapid local heating to 39°C (Roberts et al., 2017). In the present case study (Turner, Stanhewicz, Nielsen, & Wong, 2023), maximal CVC shows a similar CV (20.4–20.6%). While it is possible the presented data reflect typical variation in maximal CVC between sites in young adults, it is also important to note that CV of maximal CVC has not been established for women specifically, either naturally cycling or using OCP. Therefore, it is possible that women overall, or subgroups of women, may display different ranges or distributions in maximal CVC than previously observed in young men (Roberts et al., 2017). Regardless of possible interpretative discrepancy between absolute values and values normalized to maximal CVC, normalizing these values is crucial for making comparisons between sites within the same individual, across different time points, or between separate groups of individuals (Johnson et al., 2014). We also recognize that there are limitations to laser‐Doppler flowmetry. Because we do not know the blood velocity or the area, red blood cell flux provides an index of blood flow but may not be completely equivalent to actual skin blood flow; however, these data do provide some foundation for our understanding of microvascular physiology in premenopausal women.

Kirby et al. make another important point regarding interpretations of plateau data in general versus the nitric oxide (NO) contribution to the heating response specifically. There are several mechanistic pathways that contribute to the overall cutaneous vasodilatation response to local heating, including NO, endothelium‐derived hyperpolarizing factors, inflammation and oxidative stress pathways. Additional research suggests that the finding from Minson et al. in 2001 of ∼70% NO contribution (Minson et al., 2001) is not representative of all participant demographics, such as non‐Hispanic Black young adults (Turner, Hayat et al., 2023) or naturally cycling women during menstruation (Turner, Stanhewicz, Nielsen, Otis et al., 2023), suggesting that there is a complex interplay in mechanisms that result in a given percentage of maximal CVC in response to a local heat stimulus. For these reasons, we would suggest that it is important to assess a general measure of endothelial function (i.e., plateau) in addition to the NO contribution. For instance, previous work shows current OCP users have greater endothelial NO synthase expression in skin and a greater reliance on NO than non‐users, but there is no evidence of greater overall vasodilator responses in those groups (Cherney et al., 2007; John et al., 2000). In relation to the present work (Turner, Stanhewicz, Nielsen, & Wong, 2023), does a robust NO contribution negate the potential implications of a significantly lower standardized blood flow response to a set stimulus? This represents one of many important questions that require further discussion and investigation.

Finally, Kirby et al. refer to the contradictory nature of epidemiological data and OCP use, where the literature suggests current OCP users have increased risk of cardiovascular and thromboembolic events acutely, but former OCP users may have some protection against atherosclerosis long‐term. We suggest these may be mediated chronologically by different mechanisms. Pregnancy outcomes may yield a more temporally adjacent context of the vascular milieu. OCP use within the past 12 months is associated with adverse pregnancy outcomes, where those who used OCP within 12 months of conception had elevated relative risk of pre‐eclampsia, pre‐term birth, and low birthweight compared with those who did not (Schreuder et al., 2023). As Kirby et al. mention as well, there may be complex social underpinnings to this phenomenon, and more targeted investigation may provide insights.

Overall, there are many factors that may contribute to vascular physiology in the context of OCP use (i.e., ethinyl oestradiol dose, progestin formulation, progestin dose, duration of use, other clinical conditions, etc.), and significant gaps in the literature remain (Williams & MacDonald, 2021). We hope the present observations and this discussion inspire questions in others and move this area of research forward. Again, we thank Kirby et al. for sharing their thoughts and feedback.

## AUTHOR CONTRIBUTIONS

Casey G. Turner drafted the manuscript; all authors provided critical edits and revisions. All authors have read and approved the final version of this manuscript and agree to be accountable for all aspects of the work in ensuring that questions related to the accuracy or integrity of any part of the work are appropriately investigated and resolved. All persons designated as authors qualify for authorship, and all those who qualify for authorship are listed.

## CONFLICT OF INTEREST

The authors declare that they have no conflicts of interest, financial or otherwise.

## FUNDING INFORMATION

No funding was obtained for this work.
